# Supplementary material for: Childhood Brain Tumors: A Review of Strategies to Translate CNS Drug Delivery to Clinical Trials
Source: Cancers (Basel). 2023 Jan 30;15(3):857. doi: 10.3390/cancers15030857 (PMC9913389; doi:10.3390/cancers15030857)
Supplement: Supplementary file 1 [file cancers-15-00857-s001.zip › cancers-2154426-supplementary.pdf]

Supplementary Material

Table S1: Summary of childhood brain tumor characteristics

Image/Presentation	Histological/Molecular/Anatomy	Treatments	Outcomes, toxicities, survival and late consequences
Medulloblastoma commonest malignant brain tumor in childhood.<br><br>Presentation: raised intra-cranial pressure, 6th nerve palsy, weight loss, head tilt, neck pain, ataxia, cognitive decline.	Medulloblastoma (15%)<br>Anatomy: Cerebellar<br><br>Staging: Localized versus metastatic<br><12 bio-characterization categories, now driving new trials.	Surgery: Tissue sampling, primary tumor resection, management of raised ICP.<br><br>CT: Adjuvant therapy in routine use enhancing survival rates.<br><br>RT: Cranio-Spinal.<br><br>Survival: 40-80% 5 year survival.	Cerebellar mutism – slowed global processing speed and ataxia.<br><br>Bone marrow toxicity, ototoxicity, renal toxicity.<br><br>Somnolence syndrome,<br>late cognitive impairment, endocrine impairment of growth and development.
Diffuse Midline Glioma (DMG)<br><br>Presentation: Progressive onset of difficulties with swallowing/choking, squint, facial palsy, head tilt or neck pain, headache, vomiting, motor weakness, unsteady gait.	Astrocytoma predominantly grade 3 & 4 (10-15%)<br><br>Anatomy: Midline structures, brainstem midbrain and thalamus.<br><br>H3 Histone mutations affecting 80%, BRAF v600E 8-10% MEK 2/1 pathway mutations.	Surgery: Biopsy and management of raised ICP.<br><br>RT: Involved field.<br><br>CT: No drug therapy effective.<br><br>Survival rates: <20% survival at 2 years.	Local bleeding causing focal neurology, somnolence syndrome.<br><br>Progressive brainstem failure leading to death.

Low Grade Glioma<br><br>Presentation: Association with NF1, progressive visual symptoms, endocrine deficiencies (growth disturbance), gait unsteadiness with headaches and vomiting.	Pilocytic astrocytoma grade 1 (<40%)<br>>70% associated with MAP kinase pathway mutations.<br><br>Ganglioglioma, PXA both associated with BRAF V600E mutations. 1/3 hypothalamic, 1/3 cerebellar remainder can occur in any brain region.<br><br>Strong association with NF1 (OPG), Tuberous Sclerosis (SEGA).	Surgery: Primary surgery where safe and if complete sole therapy, tissue sampling and management of raised ICP.<br><br>CT: Carboplatin and vincristine 1st line, vinblastine 2nd line,<br>bevacizumab 3rd line for critical neurological deterioration, i.e. vision loss.<br><br>RT: Limited role in persistently progressive and chemo-resistant tumor.<br><br>Survival: resectable >90% 5 years unresectable tumors ~50% at 5 years. OS: Overall >90% at 10 years	Focal neurological risks of surgery and tumor effects.<br><br>Bone marrow toxicity, hypersensitivity, neurotoxicity. Local hemorrhage, hypertension.<br><br>Local radiation damage to brain structures and vascularity.<br><br>Focal injuries related to tumor and its treatment.
Ependymoma<br><br>Presentation: in infancy and early childhood. Symptoms of raised ICP, focal brain from cortical or brainstem regions, in later life association with multiple spinal ependymomas with NF2.	Histology: Immunohistochemical and genomic markers identifying 9 subgroups in posterior fossa (PF), supratentorial (ST) and spinal locations.	Surgery: Primary surgery for tissue sampling, debulking and management of raised ICP. Where surgery is complete it can be curative in certain subtypes – see below. Posterior fossa tumors are often intimately involved in brainstem structures. Supratentorial tumors whilst often large are amenable to extensive debulking, 50% of cases result in incomplete surgical resection.<br><br>RT: Offered to those with incomplete resection and can be deferred until the child is older or if the field is to be extensive.<br><br>CT: Trials are in progress to identify effective agents.<br><br>Prognosis is determined by molecular/anatomical subtypes with ST Yap and PF EPN-B having the best outcomes (>90% 5 year survival) and ST RELA and PF EPN-A having poorer prognosis (~50% 5-year survival).	Focal neurological injury from multiple surgeries. PF tumors in particular can lead to bulbar palsies need for tracheostomy.<br><br>RT can lead to growth failure, endocrine and cognitive damage when used in early life.<br><br>Spinal cord tumors threaten para-paresis and paralysis.

Atypical Teratoid Rhabdoid Tumor (ATRT)<br><br>Presentation: Infancy with disturbance of development, accelerated head growth, symptoms of raised ICP and focal neurology, can arise in posterior fossa or supra-tentorial region metastasis common.	Histology: Requires INI mutation and identification of SMARCB1 and 4 mutations. EZH and SNF5 are also associated.	Surgery: for tissue sampling and management of raised ICP. CSF sampling and neuraxis imaging required to identify metastasis which are common at diagnosis and present on therapy.<br><br>RT: Used in older patients and contributes to disease control.<br><br>CT: Intensified combined chemotherapy with sarcoma-like protocols show promise. Intrathecal therapy is in use to control metastasis. Loss of dose intensity can result in disease recurrence. New biotargets are under investigation in trials.	Survival occurs in fewer than 50%. Intensified CT can lead to hearing, renal, cardiac and bone marrow toxicities.<br><br>Irradiation to the developing brain in early life can lead to global brain injury and inevitable consequences.
Embryonal tumor with multi-layered rosettes (ETMR). Rapidly developing tumors most common in infancy and early childhood. Presenting with rapid head growth developmental delay, symptoms of raised ICP and progressive neurology.	Previously referred to as supratentorial PNET/high grade glioma in childhood. Now recognized in new category characterized by amplification at 19q13.42 associated functionally with diffuse expression of the LIN28A protein.	Surgery: Tissues sampling, debulking and management of raised ICP.<br><br>RT: Irradiation of primary and metastatic disease.<br><br>CT: Intensified approaches of multimodal therapy has produced improved survival. Molecular characteristics are yet to be tested in clinical trials.	Survival occurs in fewer than 50%. Intensified CT can lead to hearing, renal, cardiac and bone marrow toxicities.<br><br>Irradiation to the developing brain in early life can lead to global brain injury and inevitable consequences.

Abbreviations: PFS, progression free survival; OS, overall survival; ICP, intercranial pressure; CT, chemotherapy; RT, Radiotherapy; CSF, cerebrospinal fluid.

Table S2: Current neuro-interventional clinical trials in intra-arterial chemotherapy

Indication	IA Agent(s)	NCT Number	Timeline and Enrolment Status
**Ocular Retinoblastoma**	Multiple	NCT00901238	2009-2010 Completed
**Ocular Retinoblastoma**	Multiple	NCT03267459	2018-2025 Recruiting
**Ocular Retinoblastoma**	Melphalan	NCT01393769	2011-2016 Terminated (low enrolment in rare disease)
**Ocular Retinoblastoma**	Melphalan	NCT01293539	2011-2017 Terminated (IAC now considered standard of care)
**Diffuse Intrinsic Pontine Glioma**	Melphalan	NCT01688401	2012-2017 Suspended (interim review)
**Ocular Retinoblastoma**	Not Specified	NCT01151748	2010-2012 Withdrawn (IND agreement not reached)
**Ocular Retinoblastoma**	Multiple	NCT02955524	2016-2020 Not yet recruiting
**Ocular Retinoblastoma**	Melphalan	NCT00906113	2011-2017 Recruiting
**Ocular Retinoblastoma**	Melphalan	NCT02097134	2014-2018 Active, not recruiting
**Ocular Retinoblastoma**	Melphalan, Carboplatin	NCT00857519	2009-2016 Completed
**Ocular Retinoblastoma**	Multiple	NCT03450590	2018-2019 Active, not yet recruiting
**Ocular Retinoblastoma**	Multiple	NCT02866136	2016-2027 Recruiting
**Ocular Retinoblastoma**	Multiple	NCT02116959	2014-2019 Active, not recruiting
**Brain Metastases**	Carboplatin	NCT00362817	2004-2008 Completed
**Malignant Glioma**	Carboplatin	NCT00075387	2003-2021 Recruiting

**Malignant Glioma**	ADV-TK Adenovirus	NCT00870181	2008-2012 Completed
**Malignant Glioma**	Cetuximab (BBBD with Mannitol)	NCT02861898	2016-2019 Recruiting
**Malignant Glioma**	Melphalan (BBBD with Mannitol)	NCT00253721	1998-2016 Terminated (Other competing clinical trials affecting enrolment)
**Malignant Glioma**	Cetuximab (BBBD with Mannitol)	NCT02800486	2016-2021 Recruiting
**Malignant Glioma**	Bevacizumab	NCT02285959	2014-2020 Recruiting
**Malignant Glioma**	Temozolomide	NCT01180816	2010-2018 Active, not recruiting
**Malignant Glioma**	Bevacizumab	NCT00968240	2009-2014 Completed
**Malignant Glioma**	Cetuximab (BBBD with Mannitol)	NCT01238237	2009-2016 Completed
**Breast Cancer Brain Metastases**	Trastuzumab	NCT02571530	2015-2020 Recruiting
**Glioma**	Cetuximab, Bevacizumab	NCT01884740	2013-2019 Recruiting
**Malignant Glioma**	Bevacizumab	NCT01811498	2013-2020 Recruiting
**Malignant Glioma**	Bevacizumab, Carboplatin	NCT01386710	2011-2020 Suspended (PI transferring to new institution)
**Malignant Glioma**	Bevacizumab	NCT01269853	2010-2018 Recruiting
**Vestibular Schwannoma**	Bevacizumab	NCT01083966	2011-2020 Suspended (PI transferring to new institution)
**CNS Embryonal or Germ Cell Tumors**	Melphalan, Carboplatin (BBBD with Mannitol)	NCT00983398	2009-2020 Recruiting

(Table S2 reprinted with permission under a Elsevier and Copyright Clearance Center License; License No. 5472700032068

Table S3: Published clinical trials and clinical reports of CED in high grade glioma

**Trial design/number of patients**	**Patient group**	**Infused drug**	**Catheter used**	**Outcome**
Phase I concentration escalation (18 patients)	Adults. Recurrent malignant glioma	Transferrin-CRM107	Sialastic peritoneal CSF shunt catheter	Local toxicity > 1μg/mL<br>Local disease regression in 2 patients
Phase I feasibility (9 patients)	Adults: recurrent high grade glioma	IL-4 Pseudomonas exotoxin	CSF shunt catheter	Local tumor necrosis requiring craniotomy in 6/9 patients. 1 long term survivor.
Phase I concentration escalation (31 patients)	Adults: recurrent high grade glioma	IL-4 Pseudomonas exotoxin	CSF shunt catheter	Maximum tolerated dose 6 μg/mL. Median survival 8.2 months
Phase I feasibility (8 patients)	Adults: High grade glioma	HSV-1-*tk* gene liposome	CSF shunt catheter	Feasibility study

Phase I/II (15 patients)	Adults: recurrent high grade glioma	Paclitaxel	CSF shunt catheter	40% infusions caused toxicity. 13% severe infection rate. Suggestion of efficacy in 2 patients
Phase I/II (52 patients)	Adults: newly diagnosed and recurrent high grade glioma	Cotara (^{131}I-chTNT-1/B MAb)	CSF shunt catheter	25% procedural complications. Median survival 37 weeks (recurrent glioblastoma patients).
Phase I (51 patients)	Adults: recurrent high grade glioma	IL-13 pseudo toxin (IL13-PE38QQR)	CSF shunt catheter.	Maximum tolerated concentration 0.5 µg/mL. No distribution analysis
Phase I (22 patients)	Adults: recurrent high grade glioma	IL13-PE38QQR	CSF shunt catheter.	Maximum tolerated concentration 0.5 µg/mL. No distribution analysis
Phase I (20 patients)	Adults: recurrent high grade glioma	TP-38. (pseudomonas	CSF shunt catheter.	Maximum tolerated dose 100ng/mL Poor drug distribution with reflux into CSF spaces.

		exotoxin bound to TGFα)		
Phase III vs Gliadel wafers (276 patients. 183 in CED arm)	Adults. Recurrent high grade glioma	IL13-PE38QQR	CSF shunt catheter.	Poor drug distribution. No survival benefit demonstrated
Randomized phase IIb (145 patients)	Adults. Recurrent high grade glioma	Trabedersen (TGFβ2 antisense oligonucleotide)	External ventricular drain connected to a subcutaneous reservoir	No effect – poor trial design. No distribution analysis.
Phase Ib. (16 patients)	Adults: recurrent high grade glioma	Topotecan	External ventricular drain	Maximum tolerated dose 0.1mg/mL. Suggestion of efficacy in 4 of 16 patients
Case report	Pediatric DMG	Topotecan	External ventricular drain	Survival not enhanced. Proof of concept. No toxicity.
Case report (1 patient)	Pediatric: DMG	Carboplatin	Fused silica recessed step catheter	Large volume infusion well tolerated.

Case report (1 patient)	Adult: recurrent Glioblastoma	Carboplatin	Carbothane recessed step catheter with transcutaneous port	Procedure and infusions tolerated. 3 cycles administered.
Phase I dose escalation (28 patients)	Pediatric DMG	[^{124}I]-8H9 radiolabelled antibody against B7-H3	SmartFlow cannula (MRI Interventions, Irvine, CA, USA) or SmartFlow Flex catheter (Brainlab AG, Munich, Germany)	No treatment related grade 4 toxicity. Procedure well tolerated
Phase 1 dose escalation (20 patients)	Adult: recurrent Glioblastoma	Delta24-RGD (DNX-2401) oncolytic adenovirus	Not specified	Self-limiting toxicity due to local inflammation
Single-centre, open-label, single-arm, phase 1b	Adult: recurrent Glioblastoma	Topotecan	Chronic infusion using Synchromed (Medtronic) subcutaneous pump	Chronic CED of topotecan is a potentially safe and active therapy for recurrent glioblastoma

			1·5-mm outer diameter silastic Spetzler lumbar shunt catheter	
Compassionate treatment program (8 patients)	Pediatric: DMG	Carboplatin and Sodium Valproate	Carbothane recessed step catheters with transcutaneous port (Renishaw NeuroInfuse™)	Side-effects during brainstem infusion are common, can be transient or persist longer than 24h.

Table S4: Drug mechanism references and databases

Website	Address
Drug central online drug compendium	http://drugcentral.org/
Drugbank	https://www.drugbank.ca/
Clue drug repurposing hub	https://clue.io/repurposing-app

Table S5: Approved drugs used in pediatric drug screening

Drug/Disease Model	Mode of Action/Target	Therapeutic Class	Sensitivity
Resveratrol	Cytochrome P450, SIRT	Antioxidant	
3′-Azido-3′-deoxythymidine	Nucleoside reverse transcriptase Inhibitor	Antiretroviral	
5-aza-cytidine	DNA methyltransferase inhibitor	Antineoplastic	
5-Fluorouracil	DNA synthesis inhibitors	Antineoplastic	
Actinomycin D	RNA synthesis inhibitor	Antibiotic/Antineoplastic	ETMR, MB
Albendazole	Tubulin inhibitor	Antihelminthic	
Alfuzosin	Receptor agonist/antagonist	Antineoplastic	
Amsacrine	DNA topoisomerase inhibitor	Antineoplastic	MB
AN2728	PDE4 inhibitor	Anti-inflammatory	
Ancitabine Hydrochloride	Antineoplastic	Antineoplastic	MB
Arsenic trioxide	DNA fragmentation	Antineoplastic	
Artemether	ATP1A1	Anti-malarial	
Artenimol	ENO1 and FLNA	Anti-malarial	
Artesunate	DNA synthesis inhibitors	Anti-malarial	
Atorvastatin	HMG-CoA Reductase Inhibitors	Statin	
Auranofin	Other	Anti-inflammatory	
Axitinib	RTK inhibitor	Antineoplastic	
Azacitidine	DNA methyltransferase inhibitor	Antineoplastic	
Azacytidine	DNA methyltransferase inhibitor	Antineoplastic	
Azathioprine	DNA synthesis inhibitor	Immunosuppressant /antineoplastic	
AZD 2281 (Olaparib)	PARP inhibitor	Antineoplastic	
BCNU*	DNA alkylating agent	Antineoplastic	HGG
Benzalkonium Chloride	Cationic surfactant	Antibacterial/antifungal	
Bexarotene	RXR activator	Antineoplastic	
BIBW 2992 (Afatinib)	RTK inhibitor	Antineoplastic	
Bicalutamide	Androgen receptor inhibitor	Anti-androgen	
Bortezomib	26S preoteasome	Antineoplastic	ETMR, HGG
Bosutinib	Tyrosine kinase inhibitor	Anti-neoplastic	
Cabozantinib	Tyrosine kinase inhibitor	Antineoplastic	
Cantharidin	Phosphatase inhibitor	Antineoplastic	
Capecitabine	Nucleic acid synthesis inhibitor	Antineoplastic	
Carboplatin	DNA cross linking agent	Antineoplastic	HGG
Carfilzomib	Proteasome inhibitor	Antineoplastic	HGG
Cerivastatin Na	HMG-CoA Reductase Inhibitors	Statin	
Cetylpyridinium Chloride	CASP2 inhibitor	Antibacterial/antifungal	

Ciclopirox Olamine	Protein synthesis inhibitor	Antibacterial/antifungal	
Cisplatin	Alkylating agent	Antineoplastic	
Colchicine	Microtubule assembly	Anti-inflammatory	
CP-690550 (Tofacitinib)	JAK3 inhibitor	Antirheumatic	
Crizotinib	Receptor tyrosine kinase inhibitor	Antineoplastic	HGG
Curcumin	PPARy, vit D3 receptor	Anti-neoplastic	
Cyclophosphamide	Alkylating agent	Immunosuppressant /antineoplastic	
Cyclosporin A	Phosphatase inhibitor	Immunosuppressant	
Cytarabine	DNA synthesis inhibitors	Antiviral/antineoplastic	MB
Dasitinib	BCR/ABL, c-Kit, Src, Ephrin	Antineoplastic	HGG
Daunorubicin	Topoisomerase inhibitor	Antineoplastic	ETMR
Decitabine	Inhibits methyltransferases	Antineoplastic	
Dequalinium chloride hydrate	Bacterial permeability inducer	Antibacterial	
Docetaxel	Microtubule assembly	Antineoplastic	ETMR
Doxorubicin	Topoisomerase inhibitor	Antineoplastic	ETMR, MB, HGG
Epirubicin Hydrochloride	DNA synthesis inhibitors	Antineoplastic	
Eribulin	Microtubule inhibitor	Antineoplastic	HGG
Erlotinib	Protein kinase inhibitor	Antineoplastic	
Etoposide	Topoisomerase inhibitor	Antineoplastic	ETMR, MB, HGG
Everolimus	mTOR inhibitor	Immunosuppressant /Antineoplastic	
FK-506	FKBP-12 binding protein	Immunosuppressant	
Fluphenazine dihydrochloride	Dopamine receptor antagonist	Antipsychotic	
Fostamatinib	SYK inhibitor	Anti-inflammatory	HGG
FTY720 HCl	Sphingosine 1-phosphate receptor modulator	Immunosuppressant	
Gefitinib	RTK inhibitor	Antineoplastic	
Gemcitabine HCl	Nucleic acid synthesis inhibitor	Antineoplastic	ETMR
Gentian Violet	DNA binding	Antibacterial/antifungal	
Gramicidin	Bacterial permeability inducer	Antibacterial/antifungal	
Homoharringtonine	DNA synthesis inhibitors	Antineoplastic	
Idarubicin hydrochloride	Topoisomerase inhibitor	Antibacterial /antineoplastic	MB
Ifosfamide	Alkylating agent	Immunosuppressant /antineoplastic	
Imatinib	Tyrosine kinase inhibitor	Antineoplastic	
Irinotecan	Topoisomerase inhibitor	Antineoplastic	
Itavastatin	HMG-CoA reductase inhibitors	Statin	MB
Itraconazole	Cytochrome P450	Antifungal	
Lapatinib	EGFR-ErbB-2 inhibitor	Antineoplastic	

Maraviroc	CCR5 antagonist	Antiretroviral	
Mechlorethamine	DNA synthesis inhibitors	Antineoplastic	
Melphalan	Alkylating agent	Antineoplastic	HGG
Mercaptopurine	Methyltransferase inhibitors	Antineoplastic	
Methotrexate	Nucleic acid synthesis inhibitor	Immunosuppressant /antineoplastic	
Mitoxantrone	Topoisomerase inhibitor	Antineoplastic	HGG
Montelukast sodium	LTR antagonist	Anti-asthmatic	
Mycophenolic Acid	IMPDH reversible inhibitor	Immunosuppressant /antineoplastic	MB
N-acetyl cysteine	SODM, VEGFRA, CYC	Mucolytic agent/antidote	
Nicotinamide	SIR3, SIR2, SIR5, SIR1, HCAR2, HCAR3, NADC, NNMT	Anti-inflammatory	
Nilotinib	BCR-ABL kinase inhibitor	Antineoplastic	
Olaparib	PARP inhibitor	Antineoplastic	HGG
Paclitaxel	Microtubule inhibitor	Antineoplastic	ETMR
Panobinostat	HDAC inhibitor	Antineoplastic	ETMR, HGG
Pazopanib	tyrosine kinase inhibitor	Antineoplastic	
PD0332991	CDK inhibitor	antineoplastic	
Pemetrexed Disodium	Nucleic acid synthesis inhibitor	Antineoplastic	
Podophyllotoxin	Microtubule assembly	Antiviral	
Ponatinib	pan-Bcr-Abl inhibitor	Antineoplastic	
Prednisone	GCR, S28A1, ANXA1	Anti-inflammatory	
Procarbazine	Alkylating agent	Antineoplastic	
Pyrithione Zinc	Other	Antibacterial/antifungal	
Quinacrine	Phospholipase A2 inhibitor	Antihelminthic	
Raltegravir	HIV integrade inhibitor	Antiretroviral	
Raltitrexed	DNA synthesis inhibitors	Antineoplastic	
Rapamycin	mTOR inhibitor	Antifungal, Immunosuppressant	
Repaglinide	Other	Antihyperglycemic	
Retinoic acid p-hydroxyanilide	Other	Antineoplastic	
Revlimid	CRBN and TNFA	Antineoplastic	
Rofecoxib	COX-2 inhibitor	Anti-inflammatory	
Ruxolitinib	Kinase inhibitor	Antineoplastic	
Simvastatin	HMG-CoA reductase inhibitors	Statin	
Sodium aurothiomalate	AA3R, EGFR, FYN, TRXR1	Anti-inflammatory	
Sorafenib	Raf, PDGFR, VEGFR, c-Kit	Antineoplastic	HGG
Spaglumic Acid	Mast cell stabilizer	Neurotransmitter	
Sunitinib	VEGFR2 and PDGFR (tyrosine kinase inhibitor)	Antineoplastic	
Tamoxifen	Estrogen receptor modulator	Antineoplastic	

Temozolomide*	Alkylating agent	Antineoplastic	
Temsirolimus	mTOR inhibitor	Immunosuppressant /antineoplastic	
Thioguanine	DNA synthesis inhibitors	Antineoplastic	
Thiotepa	Alkylating agent	Immunosuppressant /antineoplastic	HGG
Topotecan	Topoisomerase inhibitor	Antineoplastic	ETMR
Tretinoin	Retinoid receptor agonist	Antineoplastic	
Triamcinolone diacetate	lysyl oxidase antagonist	Anti-inflammatory	
Valproic acid	GSK, GABA, HDAC inhibitor	Antiepileptic	
Vandetanib	EGFR, VEGR, Ret	Antineoplastic	HGG
Veliparib	PARP inhibitor	Antineoplastic	
Vemurafenib	RAF inhibitor	Antineoplastic	
Vincristine	Microtubule inhibitor	Antineoplastic	ETMR
Vindesine Sulfate	Microtubule assembly	Antineoplastic	
Vinorelbine Bitatrate	Microtubule assembly	Antineoplastic	
Vismodegib	Hedgehog pathway inhibitor	Antineoplastic	
Vorinostat	HDAC inhibitor	Antineoplastic	HGG

Table S6: Drugs graded by eligibility for trial by intrathecal administration for medulloblastoma.

**Agents in trial**	Liposomal cytarabine (Depocyte), withdrawn from production by manufacturer<br><br>Mafosfamide, withdrawn from production by manufacturer
**Agents suitable for clinical trial**	Carboplatin<br><br>Etoposide<br><br>Nimustine hydrochloride
**Drugs requiring further investigation before clinical trial**	Floxuridine<br><br>4-hydroperoxy-cyclophosphamide
**Drugs requiring further investigation (low priority)**	Diaziquone<br><br>Mercaptopurine<br><br>Rubitecan<br><br>Topotecan<br><br>Radioimmunotherapy ^{131}I-3F8 murine monoclonal antibody
**Drugs with insufficient information to grade**	Temozolomide
